# Acute respiratory failure as a first manifestation of syringomyelia

**DOI:** 10.4103/0970-2113.63614

**Published:** 2010

**Authors:** Ali Al Bashapshe, Harsha Bhatia, Shahid Aziz

**Affiliations:** *Department of Pulmonology, Aseer Central Hospital and King Khalid University, Abha, Saudi Arabia*; 1 *Department of Neurology, Aseer Central Hospital and King Khalid University, Abha, Saudi Arabia*; 2 *Department of Medicine, Aseer Central Hospital and King Khalid University, Abha, Saudi Arabia*

**Keywords:** Respiratory failure, syringomyelia, syrinx, hypercapnia

## Abstract

A 40 year old woman presented with a short history of acute onset of breathlessness to the ER of our hospital and after initial evaluation for acute pulmonary embolism which was ruled out after carrying out the appropriate investigations, she was diagnosed to be afflicted with syringomyelia based on her neurological symptoms and clinical findings, which was confirmed by doing an MRI scan, which was her basic diagnosis that was complicated by acute hypercapnic respiratory failure. This case is being reported to highlight syringomyelia as an unusual cause of acute respiratory failure, which manifested clinically in this patient as its first presentation and the underlying neurological diagnosis has been found to be present in very few reported cases (less than 0.01% of case reports) in the available literature as the basic disease in the absence of its classical presenting features. Problems associated with acute respiratory failure in the setting of syringomyelia are discussed.

## INTRODUCTION

Syringomyelia associated with Arnold Chiari malformation may present as acute respiratory failure in less than 0.01% of cases. We report the case of a young, previously asymptomatic female who presented with respiratory distress and carbon dioxide retention with metabolic encephalopathy and was found to have extensive syrinx from high cervical to dorsal cord region with myelomalacia, without Chiari malformation.

## CASE REPORT

**Figure 1:**
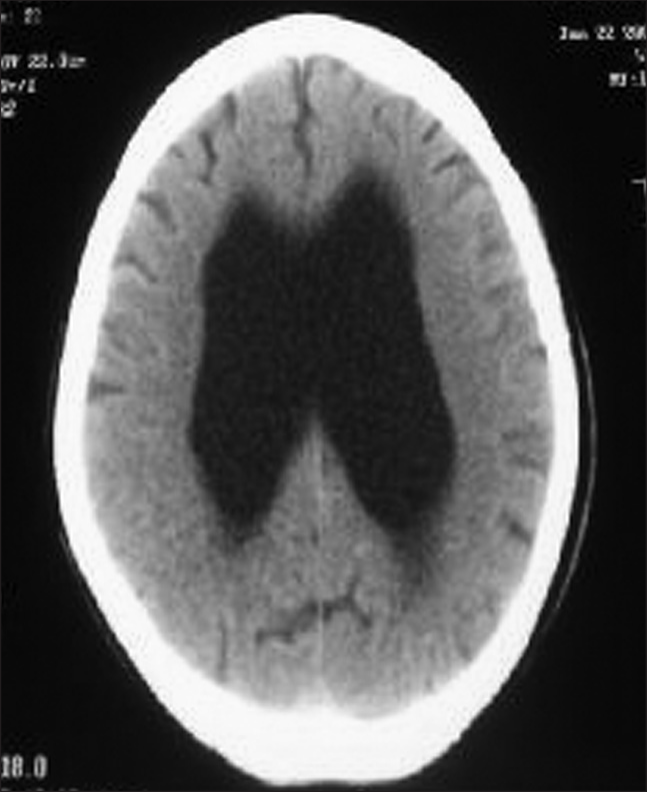
Scan head showing lobar holoproenscephaly

A 40-year-old, mother of five, presented, with a history of difficulty in breathing, of two days duration, to a peripheral hospital. On the basis of high index of clinical suspicion together with a positive serum D-dimer test she was diagnosed with pulmonary embolism. There she was started on anti-coagulation treatment accordingly, but her condition progressively deteriorated and due to worsening in her overall clinical status she was brought to our hospital. When she arrived at our Emergency Department, on the third day of her illness, she was found to be comatosed, pupils were 2 mm and reactive to light, she had roving eye movements, flaccid weakness, bilateral extensor plantars with brisk deep tendon reflexes. As she was gasping, with poor respiratory efforts and a respiratory rate of only four breaths /min, after initial assessment in the emergency room she was intubated and started on mechanical ventilation using assist control mode. Other respiratory examination was unremarkable and the focused systemic examination (other than neurological) was also non-contributory. The cranial CT scan showed holoprosencephaly of lobar type [Figure 1].

A spiral CT chest showed normal results with no evidence of pulmonary embolism. ABG done at an inspired oxygen concentration of 0.5 (FiO_2_ of 50%) showed - an arterial pH of 7.16, PaCO_2_ (arterial tension of carbon monoxide) of 78.6mmHg, PaO_2_ (arterial tension Oxygen) of 79 mmHg and a serum bicarbonate concentration of HCO_3_ 22.5mmol/L with the interpretation that with an acute respiratory acidosis mixed with metabolic acidosis, her oxygen saturation (by pulse oximetry) was only 85%. The CSF analysis was also normal. The patient was continued on mechanical ventilation along with other necessary supportive management including prophylaxes against venous thrombo-embolism and stress ulceration and was closely observed in the intensive care unit and 24 hours post-admission she showed significant improvement in her level of sensorium. She was also breathing well and hence extubated. Twelve hours later, she became drowsy again, with poor respiratory efforts and, had to be re-intubated and mechanically ventilated. During review, of her neurological status, she was noticed to have wasting of the small muscles of both her hands with brisk upper limb reflexes and decreased sensation (to pin-prick) over both arms, forearms and anterior chest wall till D6 dermatome. Power in all the limbs was grade 4 .

**Figure 2:**
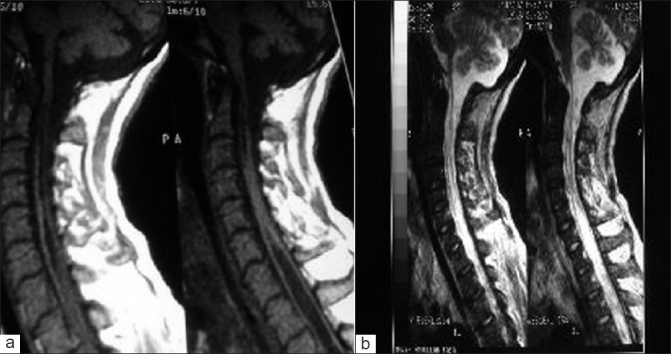
(a,b) MRI of the cervical spine saggital view showing syrinx extending from lower part of medulla to mid-dorsal region

This acute hypercapnic respiratory failure was attributed to high cervical cord lesion. The patient's Magnetic Resonance Imaging (MRI) of cervicodorsal spine was showed extensive syrinx starting from the lower medulla extending to the dorsal cord region, with myelomalacia at the cervical region [Figure 2]. Due to prolonged ventilatory requirement, she underwent a tracheostomy and could not be weaned off mechanical ventilator (she had repeated failure of weaning trials), moreover attempted temporary decannulations of tracheostomy tract caused clinically significant arterial hypoxemia. A neurosurgical consultation was taken, for definitive management, but because of the extensiveness of the patient's syrinx and her overall clinical status the neurosurgeon refused to intervene in the definitive surgical management of the patient.

## DISCUSSION

Syringomyelia (from the Greek syrinx "pipe"), may be defined as a chronic progressive neuro-degenerative or developmental disorder of the spinal cord characterized by painless weakness and wasting of the hands and arms and segmental sensory loss of the dissociated type. Pathologically there is a cavitation of the central part of the spinal cord, usually in the cervical region, extending upwards in some cases into the medulla oblongata. The onset is usually insidious and the course of this illness is unpredictable and in fact irregularly progressive, rarely there is almost an apoplectic onset or worsening.[1] In the medical literature there are very few case reports of syringomyelia presenting with an initial manifestation in the form of either acute or chronic respiratory failure![2‐4] Breathing is an essentially automatic process governed by the respiratory centres of the brainstem, which receives information on metabolic status from the peripheral chemoreceptors *via* afferent nerve fibres, and projects efferent fibres to the motor nuclei inervating the respiratory muscles. This automatic respiratory mechanism is subject to continual voluntary intervention in the waking state, but not during sleep, when the anatomical and functional integrity of the circuits is crucial. Arnold-Chiari malformation, whether alone or in combination with syringomyelia, can give rise to a variety of sudden or progressive respiratory disorders, including central alveolar hypoventilation sleep apnoea and acute respiratory insufficiency brought on by aspiration in dysphagic patients.

Our case is a rare presentation of lobar holoprosencephaly with syringomyelia manifesting as hypercapnic respiratory failure. All the other case reports of syringomyelia (syrinx) mentioned in the literature till present date, have been reported as sleep-related disordered breathing pattern in patients of syringomyelia in association with Arnold Chiari malformation or Platybasia or Basilar impression with syringomyelia. Acute respiratory failure secondary to cranio-cervical malformations should be taken into account, after all important pulmonary reasons have been ruled out, as this is a potentially treatable cause.

## CONCLUSION

Although it is very rare to present with respiratory failure, syringomyelia should be kept in mind, in such a scenario, if the clinical presentation is supporting the disease and other reasons for pulmonary symptoms have been excluded, as it is a potentially treatable condition.
